# Building health system resilience and pandemic preparedness using wastewater-based epidemiology from SARS-CoV-2 monitoring in Bengaluru, India

**DOI:** 10.3389/fpubh.2023.1064793

**Published:** 2023-02-24

**Authors:** Angela Chaudhuri, Aditya Pangaria, Chhavi Sodhi, Nitish Kumar V, Shirish Harshe, Neha Parikh, Varsha Shridhar

**Affiliations:** ^1^Swasti, Bengaluru, India; ^2^Catalyst Management Services, Bengaluru, India; ^3^Molecular Solutions Care Health LLP, Bengaluru, India

**Keywords:** COVID-19, early warning signals, epidemiological curve, inflection points, health equity, wastewater-based epidemiology, environmental surveillance

## Abstract

The COVID-19 pandemic was a watershed event for wastewater-based epidemiology (WBE). It highlighted the inability of existing disease surveillance systems to provide sufficient forewarning to governments on the existing stage and scale of disease spread and underscored the need for an effective early warning signaling system. Recognizing the potentiality of environmental surveillance (ES), in May 2021, COVIDActionCollaborative launched the Precision Health platform. The idea was to leverage ES for equitable mapping of the disease spread in Bengaluru, India and provide early information regarding any inflection in the epidemiological curve of COVID-19. By sampling both networked and non-networked sewage systems in the city, the platform used ES for both equitable and comprehensive surveillance of the population to derive precise information on the existing stage of disease maturity across communities and estimate the scale of the approaching threat. This was in contrast to clinical surveillance, which during the peak of the COVID-19 pandemic in Bengaluru excluded a significant proportion of poor and vulnerable communities from its ambit of representation. The article presents the findings of a sense-making tool which the platform developed for interpreting emerging signals from wastewater data to map disease progression and identifying the inflection points in the epidemiological curve. Thus, the platform accurately generated early warning signals on disease escalation and disseminated it to the government and the general public. This information enabled concerned audiences to implement preventive measures in advance and effectively plan their next steps for improved disease management.

## 1. Introduction

The SARS-CoV-2 pandemic proved to be a watershed event for wastewater-based epidemiology (WBE) (1–4). The rapid spread of the disease was unanticipated by conventional surveillance systems and highlighted the need for an effective early warning disease monitoring system.

Environmental Surveillance (ES) was first used for detecting poliovirus in municipal wastewaters in the 1930s (5). Its ability to detect the presence of pathogenic bacteria, virus and other microorganisms in sewage waters even during the initial stages of disease growth has been critical toward building a greater understanding of the disease transmission risks that exist in our midst (6, 7). Early on during the COVID-19 pandemic, it was established that certain people infected with SARS-CoV-2—both clinical (including pre-symptomatic) and subclinical cases—were shedding the virus RNA in their stool (6–11). This information prompted public health researchers and authorities the world over to leverage the technology for identifying and quantifying the presence of SARS-CoV-2 virus in municipal wastewaters (7–14). Since then, a number of studies have also gone on to establish a correlation between the extent of virus RNA found in wastewater and clinical case counts, thereby allowing epidemiologists to draw a progressive epidemiological curve of the virus and study temporal variations in its movement within and across communities (15).

Available evidence has shown that ES has the ability to offer a comprehensive view of the prevalence of disease in the community (7) and is capable of establishing the presence of the disease in geographies wherein clinical case data might be an under-representation of the disease numbers (16, 17). Clinical case counts are reliant on population-level reporting of disease symptoms and hence, frequently miss out on asymptomatic and mildly symptomatic disease cases. Further, they are predicated on system-level availability of diagnostic services (18). For surveillance purposes, however, the completeness of reported data in real-time is crucial (7, 19). With ES, a single pooled sample taken from sewage waters is capable of representing a relatively large cluster of people. Spatial scales can be adjusted from a few hundred persons to over a hundred thousand and even million people, depending upon the level of granularity required for surveillance (16, 20). Being an indicative surveillance tool, ES data helps to provide evidence on the presence and levels of virus circulation in a population. Through continuous monitoring, it is possible to map the changing trends in prevalence at the larger population level and identify the current stage of disease maturity in a population. For example, a significant increase in pathogen prevalence detected in wastewaters from relatively lower levels of prevalence signifies an increasing disease incidence.

The information provided by ES can aid in devising appropriate strategies for expanding the ambit of clinical testing in populations where the possibility of outbreak is high and testing levels remain relatively lower. It is particularly useful for understanding the disease burden in communities that have historically had limited access to hospital and diagnostic facilities, either due to affordability, availability or accessibility constraints. Additionally, the easily deployable nature of the technology means that even populations living in unsewered localities, having little or no connectivity to a centralized sewage system, can be systematically monitored for disease presence, without any additional resource constraint (17, 20–22). The low-cost and efficient nature of intervention makes it an ideal choice to serve as a critical complementary surveillance tool to track disease prevalence (6).

In this paper, we explore the application of sewage surveillance in Bengaluru, an urban metropolis in India, for equitable monitoring of SARS-CoV-2 and providing early warning signals on the spread of the infection in the community. Based upon its findings, a sense-making tool for delineating the different stages in the disease epidemiological cycle has been developed, which acts as a guide to help program implementers and local public health agencies to interpret the findings and insights in an informed manner and initiate requisite preventive measures.

## 2. Precision Health: An equity-focussed environmental surveillance platform

### 2.1. Epidemiological context for setting up an ES platform

From the initial stages of the COVID-19 pandemic in India till mid-2021, the government of India (GoI) and various state and city governments, including the local municipal authority in Bengaluru, relied almost exclusively on clinical data for mapping the disease trajectory. While the number of reverse transcription-polymerase chain reaction (RT-PCR) tests—the then-gold standard test for diagnosing COVID-19—was in critically short supply during the early days of the pandemic, the country bolstered its testing capacity and by April 2021, over one million tests could be conducted daily (23). However, given a population of 1.3 billion, even this increased diagnostic capacity was deemed to be insufficient as the country had some of the lowermost testing rates per 1,000 people (22, 24). Early rapid antigen test (RAT) results were blighted by poor sensitivity and specificity and the systemic reliability on RT-PCR remained.

The shortage in testing capacity in the country was exacerbated during the second wave of the pandemic—brought about by the Delta variant—as there was a significant increase in the demand for tests from the general public (25). Given the deficient testing levels, it was observed that poor, indigent and vulnerable population groups suffered disproportionately in gaining access to both diagnostic and medical care. Given their relative side-lining in the provisioning of care, there seemed to be an under-appreciation of their travails as they faced the disease burden. On the other hand, there seemed to be a labeling and stereotyping of such communities as potential disease super-spreaders, which raised questions about the non-partisan disease response by the government (26). This stigmatization precipitated testing barriers within marginalized communities as there was an element of fear of being subjected to discriminatory treatment following a positive test (27).

It was in this context, in May 2021, the COVIDActionCollaborative (CAC) initiated the Precision Health (PH) platform in Bengaluru, India. The initiative was established with the dual intent of establishing an ES-based equitable disease surveillance system in the community, which would reflect the spread of the disease among all constituent populations (as the source of data was both networked and non-networked sewage lines), without any discrimination and serve as an early disease warning signaling system for the city (28, 29).

### 2.2. Ensuring equity in site selection

With the support of the Government of Karnataka and the Bruhat Bengaluru Mahanagara Palike (BBMP), the local municipal corporation, the platform began to collect wastewater samples from 46 unsewered open drain (OD) sites in May 2021 (30). These sites were identified through a preliminary investigation of the city's drainage network and were estimated to represent a third of its population of ~12.8 million residents. These sites were located in some of the densest wards of Bengaluru city and for the most part, represented populations living in shanties situated within the city and peri-urban communities on the outskirts of Bengaluru (22). Thus, through a concerted effort, the platform ensured that some of the most socio-economically vulnerable communities in the city, who were for the most part excluded from the purview of individual testing, were given representation under PH. However, since it was difficult to identify catchment areas for different OD sites, the platform maintained its focus on serving as an integrated city-wide surveillance system.

In August 2021, the platform expanded coverage to include drainage networks connected to sewage treatment plants (STPs) within its surveillance focus and began to collect samples from an additional 28 locations (31). Altogether, the combination of networked and non-networked drainage sites represented over 80% of the city's population. The idea was to also assess if the results from either sources were comparable, since ODs have the value of immediacy but face the risk of contamination and dilution due to pollutants and the vagaries of climate, whereas watersheds of sewered connections lack the immediacy, but generally represent much larger populations and operate in a relatively more controlled environment. The hypothesis was that if the results were comparable to each other and with clinical sample trends at the overall city level, the platform would be able to draw reliable conclusions while operating in a resource-constrained environment.

### 2.3. Sample collection and processing

**Figure 1:**
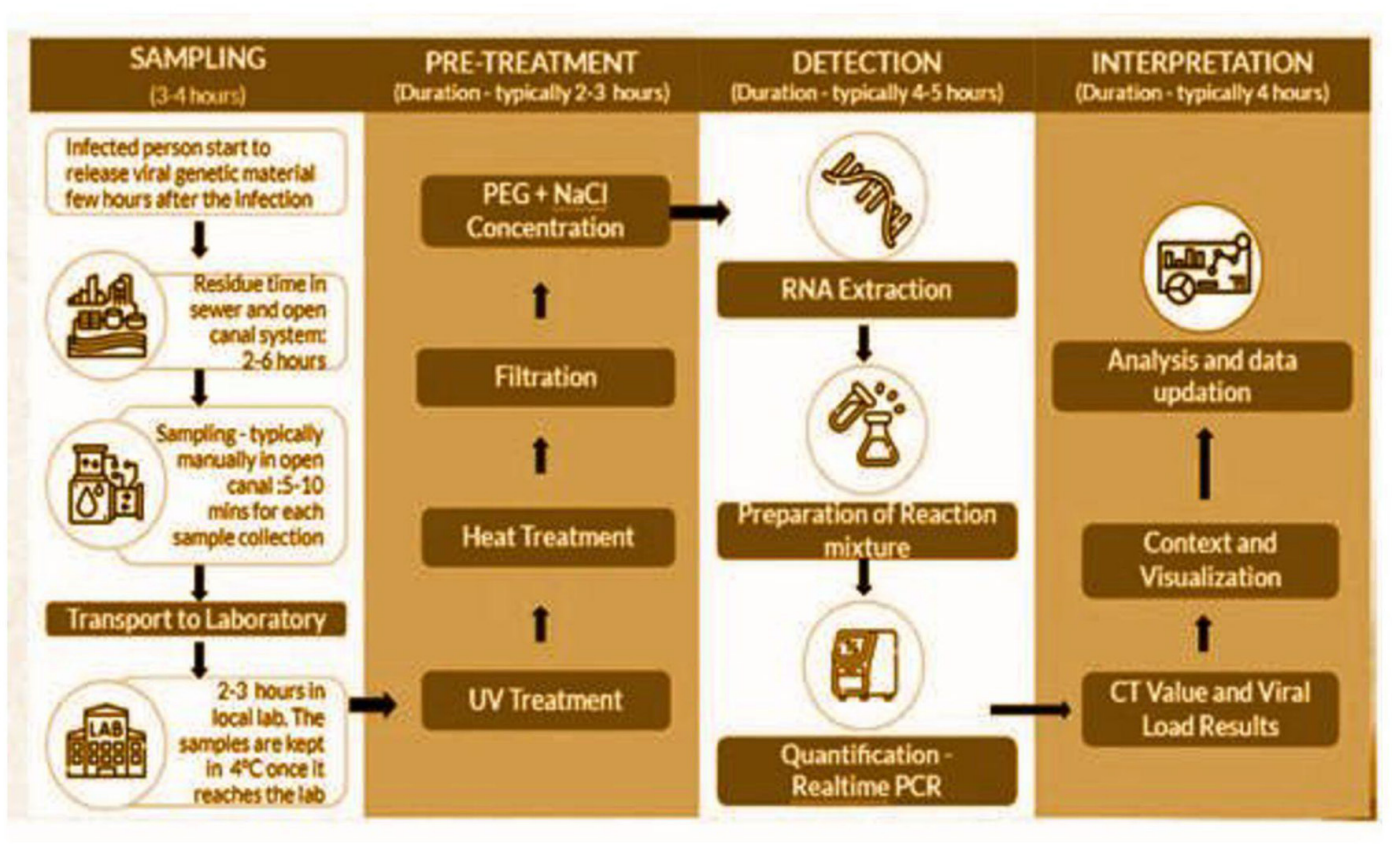
Process flow for SARS-CoV-2 testing and RNA extraction.

Samples, from each unsewered locale, are collected on a bi-weekly basis and from the different STP sites, they were collected once-a-week (using grab sampling method). They are transported to a laboratory, where, post the pre-treatment phase, they are processed to extract RNA. Subsequently, they are tested with a quantitative PCR (qPCR) kit using the real-time RT-PCR machine for quantifying viral loads (VLs). The polyethylene glycol (PEG) precipitation method, in combination with NaCl, is used for viral RNA extraction (8). The total time required in the extraction process ranged from 13 to 16 h (Figure 1)^1^.

Additionally, for genomic surveillance, eligible positive samples were also sent to a government-certified laboratory—certification provided by Indian National Accreditation Board for Testing and Calibration Laboratories (NABL), wherein they are tested using the Whole Genomic Sequencing (WGS) method.

### 2.4. Data analysis

The first item on the agenda for the platform was to measure the accuracy and consistency of wastewater data (from both OD and networked sites) by validating its findings with existing clinical data. VL and wastewater sample positivity (percent of positive samples out of the total samples tested in that week) were two indicators used by the platform for denoting trends in disease incidence. A higher presence of positive cases across the city would result in a higher VL count and greater wastewater sample positivity as the number of people shedding the virus in their stool would concomitantly increase. As people begin to shed earlier than they display disease symptoms, ES has a window of opportunity for early disease detection and short-term trend forecasting (15).

#### 2.4.1. Data equalization

For comparing wastewater and clinical positivity data trends, the platform consolidated the existing information—both daily wastewater VL values and daily clinical numbers—into weekly figures. In order to identify all early warning signals emanating from wastewater data, the platform decided to use the quantitative Exponential Weighted Moving Average (EWMA) data filter, which exponentially weighs a given data point in a time-series dataset with all the past data and helps to distinguish even slight changes in numbers from the process average. This aided the team in providing more accurate predictions on short-term disease trends (32). Under this method, more weight is given to current numbers, while the value of weights assigned to older numbers is concurrently reduced (33). The values were computed using the following formula:


(1) $$\begin{array}{r} \;EWMA=\;[a*current\;VL]\;+\;[(1-a)*\\ \left(\text{EWMA VL of the previous sample for the same site})\right] \end{array}$$

where *a* = 0.7. In our implementation, we assigned a 70% weightage to the current data points, while 30% weightage was given to older data. These weights were finalized on the basis of our programmatic learnings and under the guidance of subject matter experts.

The total wastewater samples collected by the platform from its inception till April 23, 2022 was 4,981. Sample collection from OD sites commenced from April 26, 2021, while for STP sites, collection began from August 2, 2021. Information on 58,014 total clinical samples was available for the corresponding period. Post equalization, the weekly EWMA VLs were compared with weekly clinical positivity data.

#### 2.4.2. Arriving at early warning signals

With the available wastewater data, the first order of business for the platform was to define a methodology for arriving at an early warning signal. Thus, the information generated by robust monitoring mechanisms needed to be comprehensively investigated and interpreted by program intelligence to understand its implications, else its utility would be diminished (34). Based upon its implementation learnings and increasing domain knowledge, the platform decided upon the following approach for separating the “signals” from the “noise”:

##### 2.4.2.1. Control chart limits

A univariate control chart was used, with historical EWMA VL data being applied to arrive at a centerline (represented by the mean). The upper control limit (UCL)—a horizontal line atop this centerline—was pegged at +2 standard deviation (SD). Any data variations between the centerline and UCL were considered normal, while any breach of the latter signified the need to raise a warning signal (35).

##### 2.4.2.2. Heuristics

Additionally, the platform developed a framework wherein if EWMA VLs increased by ≥25% from the previous week and this was accompanied by a rise in wastewater sample positivity rate of ≥30%, the authorities would be notified of a looming rise in disease trajectory.

### 2.5. Open data sharing for decision-making

#### 2.5.1. Local city COVID-19 war room

In Bengaluru, BBMP had set up the COVID-19 War Room, a centralized command center for managing the city's response toward the pandemic, soon after the city began to experience its first flurry of cases in 2020 (36). A central function of this body, composed of local public health officials and private sector domain experts, was to periodically review the findings of the surveillance data for improved public health planning and management.

Alongside data from clinical surveillance, the weekly data shared by PH played a key role in the decision-making process. The program intelligence unit at PH regularly interpreted the emergent signals from wastewater to highlight the existing magnitude of the problem and provide short-term forecasts on the future epidemiological curve (37). Additionally, they also shared disaggregated information on sites testing positive within the city.

#### 2.5.2. Real-time publicly viewable dashboard

The platform not only aimed at keeping local public health bodies abreast of the disease trajectory in the city, so as to enable them to implement curtailment strategies in advance, but also provided a real-time publicly viewable dashboard from a collaborative and neutral multi-stakeholder platform.

For the general populace, having reliable, continuous and importantly, equitable access to information about disease trends helped to demystify the constantly changing disease dynamics in their midst and thereby, reduce the information asymmetry vis-à-vis incidence levels, especially since numbers from clinical surveillance were not completely dependable. It was thus important for the platform to devise ways to enhance its usability in order to ensure that the available evidence was being utilized by diverse sets of people. Consultations with various types of users were used to arrive at a well-understood visualization concept. Thus, the dashboard was made color-blind friendly wherein different symbologies were used to depict shifts in measured indicators. These amendments helped to address concerns of information asymmetry by ensuring easy comprehensibility of available evidence and also enabled widespread usage and utilization of information. The information was used by clinicians, members of the scientific community, journalists reporting on COVID-19, office-administrators and office-goers for their diverse purposes. While initiating suitable preventive measures was a common corollary, the dashboard also empowered these groups to have an informed dialogue with civic agencies about measures taken.

## 3. Mapping the inflection points in epidemiological curve of COVID-19 through ES

Based upon programmatic experiences, the members of the platform developed a sense-making tool for projecting and interpreting the various stages in the epidemiological lifespan of COVID-19. As stated above, WBE helps to provide early warning signals of disease escalation (38), identify possible disease clusters for both networked and informal drainage systems (through a well-defined mapping of upstream communities) (16) and map inflection points in the disease's epidemiological curve, which are the points of most significant inference for the program and public health agencies. It was thus important to interpret and delineate the ramifications of each period and outline a potential best course of action for navigating these different junctures in the disease trajectory. We believe that this information is immensely beneficial for public health institutions and the general public, as it enables and empowers them to implement preventative measures in advance and effectively plan their next steps for improved disease management (39).

**Figure 2:**
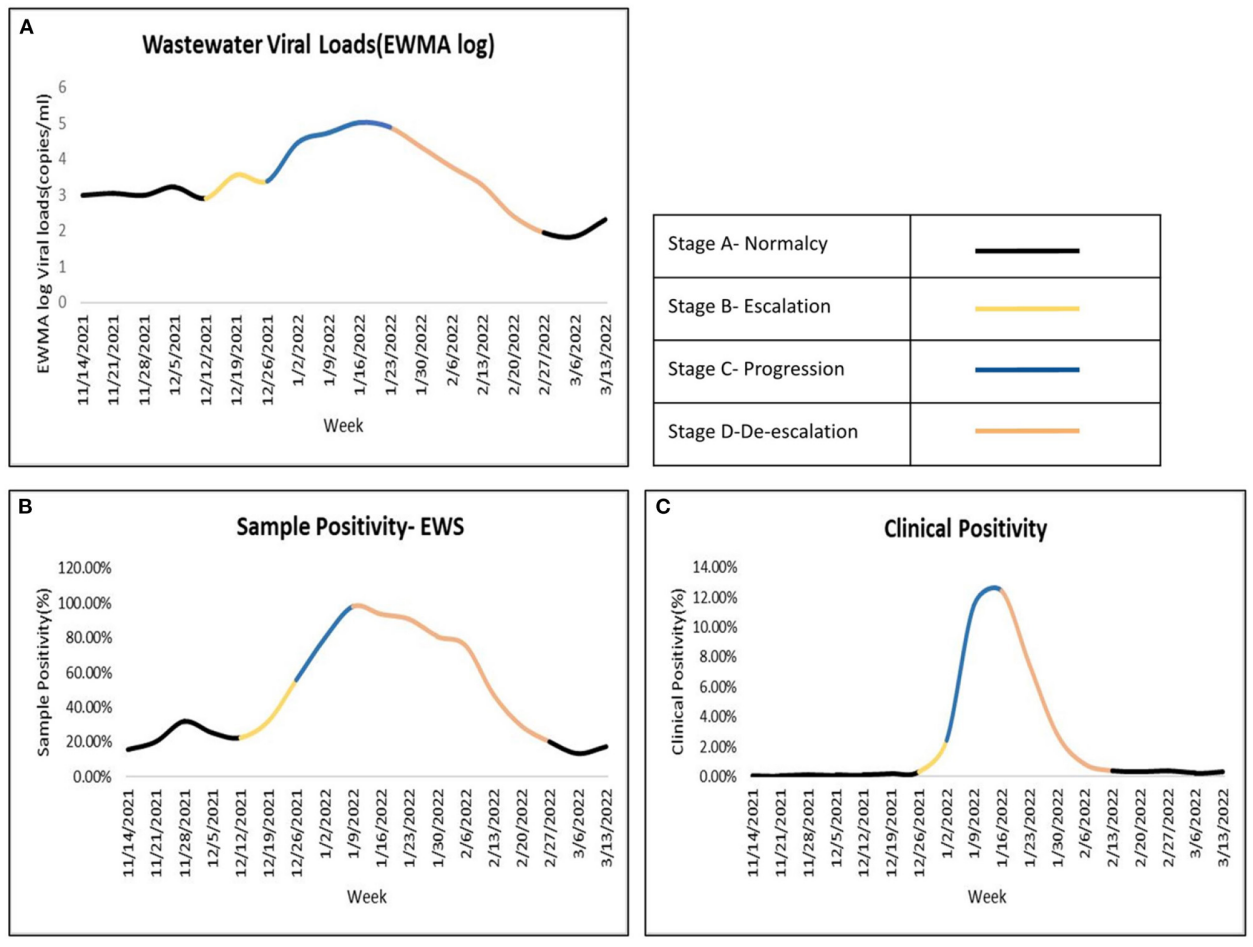
Mapping the inflection points and stages within an epidemiological curve. Top left **(A)** EWMA log of Wastewater VLs, Bottom left **(B)** Wastewater sample positivity from ES samples, Bottom right **(C)** Clinical positivity.

Figure 2 illustrates the epidemiological arc of COVID-19 as mapped through EWMA VL (Figure 2A), wastewater sample positivity (Figure 2B) and clinical positivity (Figure 2C). Using Pearson correlation coefficient, the program detected a significant correlation (*r* = 0.79, *p*-value = 9.313e-11) between the weekly EWMA viral loads and the corresponding weekly clinical positivity data for a 43-week period extending from May 15, 2021 till March 16, 2022. As evident from the figure, the inflection points in the epidemiological curve derived from wastewater sample positivity (Figure 2B) precede those derived from clinical positivity (Figure 2C), which enables ES to provide early warning signals on disease progression at the community level. In the succeeding narrative, we delve further into the major stages in the disease cycle of COVID-19, followed a brief account of the signals generated from ES at each inflection point and the concerned action points for program for leveraging the mechanism of WBE in an equitable and comprehensive manner to arrive at more precise predictions of future disease pathways. The submissions presented herein are based on our learnings from the first year of PH.

### 3.1. Stage A—Normalcy

This period is also referred to as the business-as-usual phase, as disease prevalence in the community is still quite low (see Figures 2A–C). During this underlying disease stage, there is no sustained incremental movement in incidence levels, rather there are a few random cases of disease occurring within the population. Thus, whilst monitoring wastewater samples for presence of SARS-CoV-2 at the aggregate city-level, both sample positivity and quantifiable EWMA VL values will have relatively low scores. In this stage, while there are rarely signals of disease onset, there is the possibility of a few false alarms being generated.

The platform in Bengaluru witnessed episodic fluctuations in wastewater data during this period. Utilizing existing interpretive methodologies, the team spotted early signs of disease escalation in October and November 2021, which were promptly communicated with local public health authorities for their scrutiny and further action. Upon further validation with subsequent wastewater and GS data, it was established that these were one-off spikes and not corroborated by emerging evidence.

Thus, while VLs are generally low in the collected data, it is imperative to remain alert to any set of samples with a higher-than-normative presence of the virus RNA. Even during the low-risk period, all samples eligible for GS should be sent to the concerned laboratory for decoding information on all disease variants present in the wastewater—both existing and new lineages—and mapping the presence of variants of concern (VoCs). If strains with worrisome phenotypic traits, including high transmissibility and virulence, are spotted at this stage, not only should the alert be raised but the information must be used for extrapolating future disease pathways determining the pace of change. Sentinel surveillance at major transit points, including airports, is useful at this point for augmenting information on bothersome virus strains being brought into the population.

Furthermore, the information should be triangulated with clinical data. Even a slight uptick in cases at this stage within the city or even neighboring regions can be taken as a reference to heighten surveillance vigilance. Even nascent signals should be carefully watched and used to decipher the time available before the epidemiological curve will touch its first inflection point, thereby signaling a change in the state of disease maturity. Thus, even during this relatively innocuous stage, wastewater monitoring based programs should continue being observant of all surfacing signals.

Thus, while disease outbreak indicators in this stage portend a less disquieting future of disease trajectory, there is a need to continue sustained monitoring and tracking of the disease footprint and maintain periodic communication with the government and public, whilst also flagging off any concerning signals. This period allows health authorities to focus on other population health indicators, bolster their systemic capacity to respond to future outbreaks better as well as focus on increasing vaccine coverage for the disease, particularly among vulnerable sub-groups.

### 3.2. Stage B—Escalation

The first major inflection point in the epidemiological curve is registered during this phase. In the case of COVID-19, this is the stage when ES is able to showcase a rise in the disease curvature ahead of clinical case data and generate early warning signals. For program implementers, this is the time for increasing vigil on interpreting signals from wastewater data.

This stage is typified by an increase—ordinarily a rapid spurt—in the VLs and wastewater sample positivity data gathered from ES. For PH, utilization of the aforementioned quantitative statistical techniques—control chart limits and heuristics—aided the platform to presage the onset of a new disease wave, prior to clinical data. This stage is typified by an increase—ordinarily a rapid spurt—in VLs and wastewater sample positivity. For PH, utilization of the aforementioned quantitative statistical techniques—control chart limits and heuristics—aided the platform to presage the onset of a new disease wave, prior to clinical data. In mid-December, 2021, the platform observed a 30% rise in wastewater sample positivity, alongside a >350% growth in cumulative EWMA VL values from the previous week. At this time, the number of clinical cases in the city was still low—hovering around 150–300 reported cases per day (40). On December 26, 2021, the platform, after validating the warning signs with data from one additional week, shared its early warning advisory with BBMP. Its nascent findings were subsequently validated with an increase in clinical positivity levels in the first week of January, 2022—on January 3, 2022, the city recorded >1,000 cases and over >2,000 cases the next day (40). Through GS, the platform was also able to identify the variant Omicron sub-lineage BA.2, which was primarily responsible for causing the third COVID-19 wave.

Once the first signals had emerged, it was important for the program to continue validating the existing information with subsequent data points. Based upon acquired learning, it was recognized that a mere increase in disease indicators may or may not presage the onset of a new wave. There was a possibility of cases flatlining early on without peaking. This is especially true if a large proportion of the population has been vaccinated and/or developed secondary immunity to the virus. Furthermore, while raising an early alarm, it is important to confirm the rate of disease advancement and the anticipated time available for public health agencies to initiate preventive measures, before the disease reaches its stage of full maturity. Deriving estimated infection prevalence rates at the aggregate city-level and constantly updating these numbers, based on appearing signals, is important for conveying the magnitude of the situation before the government.

The role of genomic epidemiology is critical in validating nascent signals. As we have learnt, an increase in the number of cases is typically accompanied by a greater proliferation of virus genomic lineages and sub-lineages in the wastewaters. Thus, it is recommended to focus on detecting all variants of concern (VoCs) and variants of interest (VoI) in the collected samples. Similarly, focus should be on identifying the dominant strain/s and mapping their infectivity, severity and pathogenesis (based on existing evidence) for predicting the future disease arc. At such a time, if feasible (based on the existing laboratory capacity), the frequency of sample specimens sent for GS should be increased, as its ability to provide insights into the epidemiological trajectory of the disease, even during the underlying and subclinical disease stages, is supremely beneficial for delivering more precise forecasting insights (41). It can help to clarify if the possible upsurge is being caused by more-than-one variant and transmission chains and thus, be an important guide for public health action (13).

An oft-discussed aspect of ES is its ability to identify disease hotspots at different levels of granularity (16). However, in Bengaluru, with the inclusion of informal drainage networks in our study, it proved to be difficult to identify catchment areas with complete exactness. In such a scenario, the program decided to retain its immediate focus on presenting a city-wide epidemiological understanding of the disease. Whilst selecting its sampling sites, it had ensured equitable coverage of all population groups living with the city precincts, including vulnerable population groups residing in the densely-populated inner-city areas, the various shanty-towns dotting the city landscape as well as aptly covered peri-urban communities living in outer city boroughs. Wastewater data too showed a near-simultaneous increase in EWMA VLs for both networked and non-networked areas, which underscored the need for greater focus on marginalized communities in the government's COVID-19 response, given their greater vulnerability to the disease.

For raising early warning signals for COVID-19, this is the most pivotal stage. The possibility of raising a false alarm, at this juncture, is much less costly than taking the risk of not communicating existing signals. However, as far as possible, it is important to have in place mechanisms to validate threat perceptions, so as to minimize the possibility of sounding a wrong bugle. The authorities can use these insights to take necessary action for curtailing disease spread in accordance with the severity of the situation. In a population with inadequate vaccine coverage, such early warning signals become doubly important as they enable authorities to implement preventive measures keeping in mind the differential vulnerability of various population groups. Thus, early identification allows them time to re-allocate resources, based on situational need.

Apart from the local civic agencies, it is critical to share these risk signals with the general public, who are then encouraged to institute preventive measures and in case of appearance of symptoms, get themselves tested. Similarly, office-and-business managers have used this information for bolstering protective measures at the workplaces and securing the health and safety of their workers and families.

### 3.3. Stage C—Progression

This is the peak disease stage or the stage when the disease spread is at its full maturity. The inflection point in the epidemiological curve undergoes a sharp uptick at the beginning of the phase, followed by a short plateau as the infection reaches its uppermost limit. ES data shows an increase in both VLs and wastewater sample positivity and now this increase is simultaneously accompanied by a rise in diagnosed cases as the clinical stage of the infection progression cycle is reached.

The platform, which had shared advance signals of disease intensification on December 26, 2021 with the government, had similarly forecast that infection would peak between the period of 16–19 January 2022. This was later borne by both wastewater data—wastewater sample positivity was nearly full and there was a >30-fold increase in the cumulative EWMA VL values from the former date to this peak period. Clinical data too endorsed these findings—the government announced the commencement of the third Omicron-led COVID-19 wave on January 5, 2022 (42), as the number of reported cases in the city crossed 4,000 (43). As anticipated, cases peaked on January 20, 2022, with 30,540 total fresh cases being registered on this day (44). This peak phase lasted till the end of the month in Bengaluru. The primary focus of the government at this time was on managing the disease and tending to the provisioning of adequate diagnostic and medical care at the community level.

The start of this period is critical for WBE—its role is to validate the peak phase and provide further signals that an even greater increment of disease numbers is on the anvil in the near future. Additionally, as was done by PPHS, it is important to let the authorities know the time available to them till the disease reaches its maximum peak and the estimated infection prevalence rates during those days. At this time, given that the infection has spread across the entire populace, it is important to share an aggregate city-level trend with the government. However, it is important to vet quantifiable EWMA VL trends from both open surface and formal sewerage sights to verify populations requiring greater focus.

In this stage, the role of genomic data is again especially useful. By studying the presence of VoCs and other dominant strains and mutated sub-strains in wastewater, their virulence, immune escape levels and disease pathogenesis, GS can provide critical insights for improved disease management. Additionally, it can help to serve as a guide for instituting protective population-level measures for under-vaccinated populations, including high-risk vulnerable population groups and young and adolescent children, who may still be vulnerable to the most deleterious aspects of the disease.

### 3.4. Stage D—De-escalation

This beginning of this stage marks the final inflection point in the disease arc as there is a more or less sustained downturn in incidence levels. As the virus reach is receding at the population levels, the number of new clinical cases reported too begin to decline. In Bengaluru, this point was touched on 31 January 2022, when case numbers began to decline.

At this stage, the role of ES is quite limited. Available evidence suggests that even after people recover from their COVID-19 infection, they continue to shed the virus in their excrement for a few additional weeks (33). Hence, as discovered by the program, the quantifiable EWMA VLs are prone to volatility whilst on the decline. The epidemic curve may, on occasion, depict higher prevalence levels than is the case in reality and resemble a positively skewed bell-curve. The fall in incidence levels thus needs to be triangulated with clinical data on a daily basis. It is recommended to maintain high testing rates at this point so that clinical positivity rates may help to provide more insight into the declining disease trajectory.

## 4. Discussion

The COVID-19 pandemic has highlighted the need for more efficient and representative disease monitoring mechanisms. As a community-level surveillance and early warning system, WBE has the potential to inform evidence-based decision-making ahead of normative measures of disease monitoring and substantiate the existing knowledge base of disease epidemiology, as was seen in the case of SARS-CoV-2 monitoring. Through its advanced warning capabilities, it has the ability to alert other surveillance teams about the impending threat and contribute to precautionary measures needed while dealing with and reporting suspected cases.

Historically, the problem with epidemiology has been that they have chiefly relied on clinical case counts for monitoring disease spread, which has limited its advanced prediction abilities and inhibited its representative nature. In areas with limited-service utilization and inequitable access to care, the information provided by clinical data is incomplete and exclusionary of vulnerable and indigent communities, whose access to services is the most tenuous during times of crisis. Additionally, clinical data has grappled with the problem of healthy carriers. Given the inadequacies, clinical surveillance methods have lacked the precision required to decipher early disease evolution and the stage of maturity at which a particular outbreak has been. As its methods have been more responsive than proactive, its role in restricting infection progression across populations has been limited.

At PH, the science of ES has been leveraged as a tool for equity. Inclusion of both formal and informal drainage networks in its sampling focus has not only helped to represent under-served and marginalized populations but also provided an all-embracing picture of disease scale at the wider city-level. This has aided the platform in mapping the inflection points in the epidemiological curve with greater certainty than before, giving early warning insights and also foretelling the timespan in which a contained outbreak will begin to spread at the population level. These learnings are useful for policy planners and program implementers, as they enable early application of mitigation strategies before conditions become ripe for diseases to reach maturity and aid in the design of a comprehensive disease management framework. They also help to throw light on current disease burdens across communities, particularly the indigent, which, we believe, can help to sway focus of disease management strategies to those who are most at risk of infection and vulnerable to its impact.

Ensuring equitable dissemination of emerging information has been another agenda for the platform. By providing regular access to the general public about the changing etiology and epidemiology of the disease waves, it enabled them to be proactive in applying appropriate precautionary measures and thereby, participate in saving lives of those who were vulnerable to the disease. Additionally, it facilitated an open and increasingly transparent dialogue among different constituent populations with local authorities about their role in disease mitigation. Journalists reporting on the findings of the platform and the early warning signals in both English and vernacular media further served to broaden its access.

Apart from equity, accuracy in forecasting has been a key mandate for the platform. In this process of refining our understanding of outbreak spread patterns and epidemiological inflection points, the role of genomic epidemiology was critical. For diseases such as COVID-19, wherein the causative agent is relatively new, unstable, highly mutable and still being understood, the value of GS for distinguishing variants, its lineages and sub-lineages is significant. Studying different disease cycles, utilizing well-established scientific information on genotype-phenotype associations and correlating this knowledge with disease patterns emerging in Bengaluru and India for deducing the multiple inflection points within a disease curve has been pivotal to our predictive modeling. However, a limitation that the program has contended with, particularly during the earlier implementation stage, is the time lag in the availability of genomic data. Given the dynamic nature of disease progression, these delays have limited data usability for present analysis. While wastewater sample positivity and VL data has been used for providing early warning signals, genomic epidemiology has been used for validating emerging signals, understanding disease etiology and casting prediction models.

Another programmatic limitation has been the difficulty in identifying catchment areas of selected sites. This is especially so for informal drainage networks, which made disease hotspotting at the intra-city level a relatively tricky proposition. While the platform served as a city-wide surveillance system in its first year, it recognizes the need for more in-depth geospatial mapping of sewerage networks and upstream communities for identifying high-transmission and disease vulnerable spatial clusters more effectively.

Going forward, we believe that the learnings from PH's leveraging of WBE for building standard protocols for disease forecasting can be harnessed and honed by other cities not just for COVID-19, but other infectious diseases as well. The successful deployment of ES as an ethical and equitable surveillance platform (45) enables systems to capture the health concerns of more vulnerable population groups as well. Thus, it has great usability for showcasing the complete epidemiological arc of diseases which are known to have a disproportionate impact on vulnerable communities, including hepatitis, cholera and dengue. With changing environmental conditions, as endemic infectious diseases are reaching epidemic and potentially, even pandemic levels, we should be able to borrow the learnings from the above for understanding, predicting, managing, and generating effective disease mitigation strategies. This intelligence can be relevant not just for a specific country, but used globally as well.

## Data availability statement

The raw data supporting the conclusions of this article will be made available by the authors, without undue reservation.

## Author contributions

AC conceptualized the research. AP and NKV conducted the statistical analysis. CS and AP drafted the manuscript. AC, NKV, SH, NP, and CS revised and edited the final draft. All authors reviewed the manuscript.

## Footnotes

^1^For more detailed information on method used for detecting and calculating SARS-CoV-2 RNA presence in wastewater samples, please see Supplementary Data 1.
