# Supplementary material for: Building health system resilience and pandemic preparedness using wastewater-based epidemiology from SARS-CoV-2 monitoring in Bengaluru, India
Source: Front Public Health. 2023 Feb 24;11:1064793. doi: 10.3389/fpubh.2023.1064793 (PMC9999730; doi:10.3389/fpubh.2023.1064793)
Supplement: Supplementary file 1 [file Data_Sheet_1.PDF]

Building Health System Resilience and Pandemic Preparedness using wastewater-based epidemiology from SARS-CoV-2 monitoring in Bengaluru, India

Angela Chaudhuri[1*], Aditya Pangaria[2†], Chhavi Sodhi[1†], Nitish Kumar[2†], Shirish Harshe[1], Neha Parikh[1], Varsha Shridhar[3]

[1] Swasti Health Catalyst, Bengaluru, India
[2] Catalyst Management Services, Bengaluru, India
[3] Molecular Solutions Care Health

†These authors contributed equally to this work and share second authorship

*Correspondence:
angela@catalysts.org

1 Supplementary Data

Method for detecting and calculating SARS-CoV-2 RNA in wastewater

Detection and quantification of SARS-CoV-2 was carried out using the GenePathDx CoViDx One v2.1.1TK-Quantitative RT-qPCR Kit. The kit contains three concentrations (5000 copies/uL, 500 copies/uL and 50 copies/uL) of a synthetic plasmid bearing the SARS-CoV-2 loci targeted by the RT-qPCR assay, as quantitative standards. These standards are run in parallel with the unknown samples being tested. The RT-qPCR kit also includes a software tool that enables the determination of viral loads in unknown samples through a simple browser-based interface. Briefly, the software independently calculates a best fit curve by plotting logarithmic concentrations of the standards against the threshold cycle (Ct) value for each of the three SARS-CoV-2 targets - RdRp, N and E genes. The concentration of the unknown sample, for each of the three gene targets is individually computed using by correlating their Ct values to the best fit curve. Given that the standards are double stranded DNA molecules, whereas the target SARS-CoV-2 genome is a single stranded RNA molecule, the concentrations for each gene target were multiplied by a factor of two to arrive at final concentrations in the PCR. In addition, graphs of each sample were visually checked to ensure that they were sigmoidal in shape. Those samples with non-sigmoidal graphs were considered negative. All samples were also tested for RNaseP as an internal control. Only those samples that had an RNaseP value between 20 and 35 were considered valid. Final viral loads in the original sample were calculated by applying a factor calculated from the amount of sample used for testing and volume eluted after nucleic acid purification to the concentration determined by the PCR.
